# EEG correlation at a distance: A re-analysis of two studies using a machine learning approach

**DOI:** 10.12688/f1000research.17613.2

**Published:** 2019-03-29

**Authors:** Marco Bilucaglia, Luciano Pederzoli, William Giroldini, Elena Prati, Patrizio Tressoldi

**Affiliations:** 1 Behavior and Brain Lab, Università IULM, Milano, Italy; 2 EvanLab, EvanLab, Firenze, Italy; 3 Science of Consciousness Research Group, Dipartimento di Psicologia Generale, Università degli Studi di Padova, Padova, 35131, Italy

**Keywords:** EEG, correlation at distance, machine learning, linear discrimination analysis.

## Abstract

**Background:** In this paper, data from two studies relative to the relationship between the electroencephalogram (EEG) activities of two isolated and physically separated subjects were re-analyzed using machine-learning algorithms.

The first dataset comprises the data of 25 pairs of participants where one member of each pair was stimulated with a visual and an auditory 500 Hz signals of 1 second duration.

The second dataset consisted of the data of 20 pairs of participants where one member of each pair received visual and auditory stimulation lasting 1 second duration with on-off modulation at 10, 12, and 14 Hz.

**Methods and Results:** Applying a ‘linear discriminant classifier’ to the first dataset, it was possible to correctly classify 50.74% of the EEG activity of non-stimulated participants, correlated to the remote sensorial stimulation of the distant partner.

In the second dataset, the percentage of correctly classified EEG activity in the non-stimulated partners was 51.17%, 50.45% and 51.91%, respectively, for the 10, 12, and 14 Hz stimulations, with respect the condition of no stimulation in the distant partner.

**Conclusions:** The analysis of EEG activity using machine-learning algorithms has produced advances in the study of the connection between the EEG activities of the stimulated partner and the isolated distant partner, opening new insight into the possibility to devise practical application for non-conventional “mental telecommunications” between physically and sensorially separated participants.

## Introduction

The test of whether or not the brain activities of two people who are only linked mentally – and with absolutely no other form of conventional communication – are correlated is a small research field that has existed for about 50 years (see Table S1 in ( Giroldini *et al*., 2016).

Despite accumulating evidence in favor of this correlation, this phenomenon is still considered controversial because of the proposed theories to explain it, no single theory is widely accepted. This correlation excludes the possibility of it originating from information received via the sense organs, from any direct link, even internet connections (e.g. ( Jiang *et al*., 2018; Lee *et al*., 2017)) or from the electroencephalogram (EEG) activity of the stimulated partner of the pair, given their physical distance and isolation from each other.

We are left with postulating a type of quantum-like connection based on the distant mental connection between each partner ( Galli Carminati *et al*., 2017; Walach & Römer 2011; Walach *et al*., 2016), even if this connection operates on a neurophysiological level.

The most commonly used method in this field of research is that of isolating the two partners, ensuring that there is no chance of sensorially obtaining information in the usual way, and the synchronous parallel recording of their respective neurophysiological activities. Therefore, one partner is stimulated at non-regular intervals with visual and/or auditory stimuli which can be structured, e.g. images, or non-structured, such as arrangements of black and white squares, or short duration sounds at a specific frequency. The choice to present the stimulations at irregular intervals, possibly also randomly, is important because it reduces neurophysiological autoactivation due to the expectation of stimulation.

At the end of the stimulation stage, each partner’s recorded neurophysiological parameters are compared. If the non-stimulated partner’s neurophysiological output shows activity that is simultaneous with the activity in the stimulated partner, and this activity is statistically greater than during the non-stimulated periods, we can confirm that it derives from a mental connection, or at least from a means that is different from conventional electromagnetic transmission.

Regarding the type of correlation between the partners’ neural activities, a further step with respect to simply proving its existence is determining whether it is aspecific or specific; this means whether the type of observed activity – for example in the activity of the partner receiving specific sensorial stimulations, such as a visual stimulation with an on-off modulation at 10 Hz and 14 Hz – is also seen in the mentally connected partner, or if the activation is undifferentiated, for example at 10 Hz.

As would be expected, in the correlated activities of the two partners, the neurophysiological activity of the non-stimulated partner is always smaller, and it could be that classical analysis methods are unable to separate the activity signal from the background.

Beginning in the last few years, thanks to the development of artificial intelligence algorithms, different machine-learning algorithms have been applied to the analysis of EEG data ( Chai *et al*., 2017; de Carvalho *et al*., 2017; Lotte *et al*., 2007; Stober *et al*., 2016). Among their many advantages is the possibility of simultaneously analyzing many variables that can be associated with a given event in both a linear and non-linear way, revealing coactivity configurations that would otherwise be difficult to see with other techniques. For an introduction to this type of algorithms see ( Lantz, 2015).

In this study we re-analysed, using machine-learning techniques, the data of two studies aiming to more precisely identify the neurophysiological parameters of the EEG activity of non-stimulated partners correlated to those produced by their partners given specific sensorial stimulations.

The first dataset was obtained from the study of ( Giroldini *et al*., 2016). In this study, conducted using 25 pairs of participants, the stimulated partner was given a series of 128 visual and auditory stimulations of 1 second duration at 500 Hz, separated by random intervals of non-stimulation lasting from 4 to 6 seconds. Each partner’s EEG activity was simultaneously recorded using 14-channel equipment.

The second dataset was obtained testing 20 pairs of participants, by ( Giroldini *et al*., 2018). In this study the stimulated partner was given random sequences of 32 visual and auditory stimulations of 1 second duration modulated at 10, 12, or 14 Hz, while the EEG activities of each partner were simultaneously recorded.

## Methods

### First dataset

***Participants.*** Six Italian Caucasian healthy adults were chosen for the experiment, comprised of five men and one woman, with an average age of 35.5 years (SD = 8.3).

They were selected from the members of EvanLab, the private laboratory involved in this study. The criteria for their voluntary inclusion were their mutual friendship (> 10 years), and their experience in being able to maintain prolonged focused concentration, a product of their familiarity with meditation and other practices requiring control of mental activities.

All together they contributed 25 different pairs of data.

The data were collected over three non-consecutive days. The dataset (available from: http://dx.doi.org/10.6084/m9.figshare.1466876 ( Tressoldi, 2016)) includes details of pairings and the EEG data from a 14 EEG channels system.

***Statement of ethics.*** The use of experimental subjects is in accordance with ethical guidelines as outlined in the Declaration of Helsinki, and the study has been approved by the Ethical Committee of the University of Padova’s Department of General Psychology (prot. n. 63, 2012). Before taking part in the experiment, each subject gave his/her informed consent in writing after having read a description of the experiment.

***Procedure.*** Each stimulated subject received 128 audio-visual stimulations (AVS). The auditory stimulus was composed of a 500 Hz sinusoid applied through 32 Ohm Parrot ZIK® earphones at a volume of about 80 dB. The visual stimulation was from high intensity red LEDs in a 4×4 arrangement placed approximately one meter from the subject being stimulated. The subject kept his/her eyes closed because the light could easily be detected through the eyelids.

Additionally, for each subject (no matter whether stimulated or not) 128 “surrogated” stimuli were drawn (nAVS). Their onsets were placed exactly 3s before the AVS onsets in order to be discriminated from the nAVS by our classification algorithm.

All preprocessing steps were done in Matlab using the EEGLab toolbox ( Delorme & Makeig, 2004).

After filtering with a zero-phase FIR band-pass filter (2~40Hz), an ICA (Independent Component Analysis) was performed using a SOBI algorithm that, according to a previous study ( Urigüen & Garcia-Zapirain, 2015), exhibits the best performance in the detection of the artifacts.

ICs (Independent Components) corresponding to artifacts were automatically identified and rejected using the MARA algorithm ( Winkler *et al*., 2011) that is based on a set of spatial, temporal and statistical features. Finally, a CAR (Common Average Reference) was performed.

Given that the type of stimulation produced a P300-like wave, we followed the common procedures used with Brain Computer Interfaces based on P300 ( Hoffmann *et al*., 2008). Data were first epoched with length of a 1s after the stimulus onset, then a sixth order Butterworth bandpass filter (1 ~ 12Hz) was applied and downsampled to 32Hz. Epoch lengths were thus lowered to 64 samples located at time points:

$${\left\{t_{k}\right\}}_{k=1}^{64}=\left\{0s,\;-0.6667s,\ldots ,+\;1.2333s,+1.2667s\right\}.$$

Samples were then amplitude-scaled according to the maximum and the minimum value inside each epoch, according to the equation:

$$x_{scaled}=\frac{x-\frac{\left(x_{max}+x_{min}\right)}{2}}{\frac{\left(x_{max}–x_{min}\right)}{2}}$$

Finally, scaled samples were concatenated to form a feature vector *v*, with a dimensionality of 65 × 14 = 910:

$$v=\left[x_{1}(t_{1}),x_{1}(t_{2}),\ldots ,\;x_{1}(t_{64}),\ldots \;x_{k}(t_{j}),\ldots \;x_{14}(t_{1}),x_{14}(t_{2}),\ldots \;x_{14}(t_{64})\right]$$

where *x _k_*( *t _j_*) is the sample at time point *t _j_* from channel *k*.

***Feature selection.*** Reduction of the feature dimensionality is crucial to improve classification performances. A simple method for feature selection is based on the ranking of the Biserial Correlation Coefficients ( Müller *et al*., 2004). Considering a two-class dataset *D* (with just one numerical feature), the Biserial Correlation Coefficient *r* ^2^ for the feature is defined as:

$$r^{2}=\left(\frac{\sqrt{N_{A}\cdot \;N_{B}}}{N_{A}+N_{B}}\frac{m(D_{A})-m(D_{B})}{s(D)}\right)$$

where *D _A_*, *D _B_* are the subsets of the dataset *D* composed by instances that belong to class *A, B*. *m*(.) and *s*(.) are, respectively, the sample mean and standard deviation operators. *r* ^2^ score is a measure of the “discriminative power” of the feature, reflecting how much of its “variance” is “explained” by the class affiliation.

After summing the coefficients of each feature and sorting the scores in descendent order, we selected the features of which the added score came to 95% of the total score.

For each stimulated and non-stimulated group of participants, the scalp distributions of *r ^2^* coefficients (expressed as percentage, normalized to the total score) were drawn. Since each channel is associated with at most 65 features (as well as 65 *r ^2^* coefficients), coefficients (one coefficient for each channel) are calculated as mean value.

***Classification.*** As a classifier, we chose a Linear Discriminant (LD) classifier and the estimation of the classification accuracy was performed 10 times (to compensate the random variability of the estimated accuracy) by a 10-fold stratified cross-validation scheme.

We choose LD because, according to the literature ( Lotte *et al*., 2007), it has a very low computational requirement, is simple to use and generally provides good results (i.e. robustness to overfitting).

Since the feature selection is performed using information from the class labels, in order to avoid the overfitting and to train the classifier in a “fair way”, feature selection was performed at each step within the cross-validation.

In addition to the LD classifier, at each step we classify our data using a Random classifier (which gets a prediction uniformly distributed between the classes) that serves as a “baseline” to evaluate the LD classifier performance. The random classification is simply obtained randomly assigning (with uniform probability p=1/n) to each instance of the test, one of the class {C_1,C_2,…,C_n }, where n is the number of classes to be predicted.

At each cross-validation step, we calculated the mean accuracy and, finally, we calculated the mean and standard deviation of these mean accuracies.

The syntax in Matlab code is available here: https://doi.org/10.6084/m9.figshare.5132647.v6 ( Tressoldi *et al*., 2018)

### Second dataset

The dataset is composed of 14 channels EEG data obtained from 20 pairs of participants (available from: https://doi.org/10.6084/m9.figshare.5132647.v6 ( Tressoldi *et al*., 2018)).

***Participants.*** Five adults, two women and three men with an average age of 38.3 years (SD = 7.5), took part in this study. The selection criteria were their experience in mind control techniques (mainly meditation) and their mutual friendship, which we consider as essential pre-requisites for an adequate “mental and emotional connection” between the pairs. Each participant took turns in being both the stimulated partner and the non-stimulated partner with each of the others, making a total of 20 pair combinations.

***Statement of ethics.*** The use of experimental subjects is in accordance with ethical guidelines as outlined in the Declaration of Helsinki, and the study has been approved by the Ethical Committee of the University of Padova’s Department of General Psychology (prot. n. 63, 2012). Before taking part in the experiment, each subject gave his/her informed consent in writing after having read a description of the experiment

This sample size of 20 pairs of participants was estimated by setting the following parameters: statistical power = .80, a one-tailed Type I error = .05 and an expected effect size *d* = .5 for a one sample t-test (difference from a constant = 0: http://powerandsamplesize.com/Calculators/Test-l-Mean/1-Sample-1-Sided).

***Stimuli.*** Visual stimuli consisted of red light flashes at 10, 12, 14 Hz (32 stimuli for each frequency) lasting 1 s with a minimum inter-stimulus interval of 4 s, while audio stimuli consisted of a 900 Hz sine tone, modulated on-off at the flashing frequencies.

The audio modulation was performed on a 900 Hz sinusoidal carrier and applied through 32 ohm impedance earphones at a volume of about 80 dB.

The interval between the three blocks was randomly varied at between 40 and 90 seconds.

The visual stimuli were provided by an array of 16 red LEDs positioned about 30 cm from the stimulated partner’s closed eyes.

The three frequency blocks were presented randomly without repetition of the same frequency.

Additionally, for each subject (regardless of whether stimulated or not) the onsets of 32 “surrogated” stimuli (0 Hz, labelled as 255) were drawn from random positions of the entire recording: the first and the last 10 s were discarded, maintaining the same minimum inter-stimulus distance.

***Preprocessing.*** All pre-processing steps were done as for the first dataset. Data were epoched with a 2s window after each stimulus onset. From each epoch and from channel, the PSD (Power Spectral Density) was estimated with a Welch’s periodogram (1s long Hamming window with 50% of overlap) and spectral bins were normalized to the maximum value of the PSD.

Following the feature extraction procedure normally used with Brain-Computer-Interfaces systems based on SSVEP ( Müller-Putz *et al*., 2005), we concatenated spectral bins corresponding to both stimulus frequencies and their first harmonics: *F* = {10, 20; 12, 24; 14, 28} Hz.

The feature vector *v*, with a dimensionality of 6 × 14 = 84, is thus defined as:

$$v=[p_{1}(10),p_{1}(20),\;\ldots \;,p_{k}(f_{j}),\;\ldots \;,p_{14}(14),p_{14}(28)]$$

where *p _k_* ( *f*) is the spectral power at frequency *f _j_* ∈ *F* from channel *k*.

***Feature selection.*** The 4-class (10, 12, 14, 0) prediction problem was decomposed in 6 different 2-class prediction problems, creating 6 different datasets containing all the possible couples of the 4 classes: {10, 12}, {10, 14}, {10, 0}, {12, 14}, {12, 0} and {14, 0}.

Feature selection was based on the ranking of the Biserial Correlation Coefficients as with the first dataset.

***Classification.*** As with the first dataset, we used a Linear Discriminant (LD) classifier compared with a Random classifier. Again, we performed a 10-fold stratified cross-validation scheme, with feature selection inside each cross-validation step.

### Experimental hypotheses

For both datasets and in the stimulated and non-stimulated partners, we expected a higher level of correct classification of the EEG activity correlated with the visual and auditory stimulations with respect to the EEG activity correlated with the periods of non-stimulation, corresponding to the inter-stimulus intervals.

However, whereas for the stimulated partners, it is expected a great difference between these two EEG activities, we expected a much small difference, even if statistically significant, in the non-stimulated partners, given that none sensorial stimulation can be transferred from the stimulated to the non-stimulated partners. 

## Results

### First dataset

**Figure 1.**
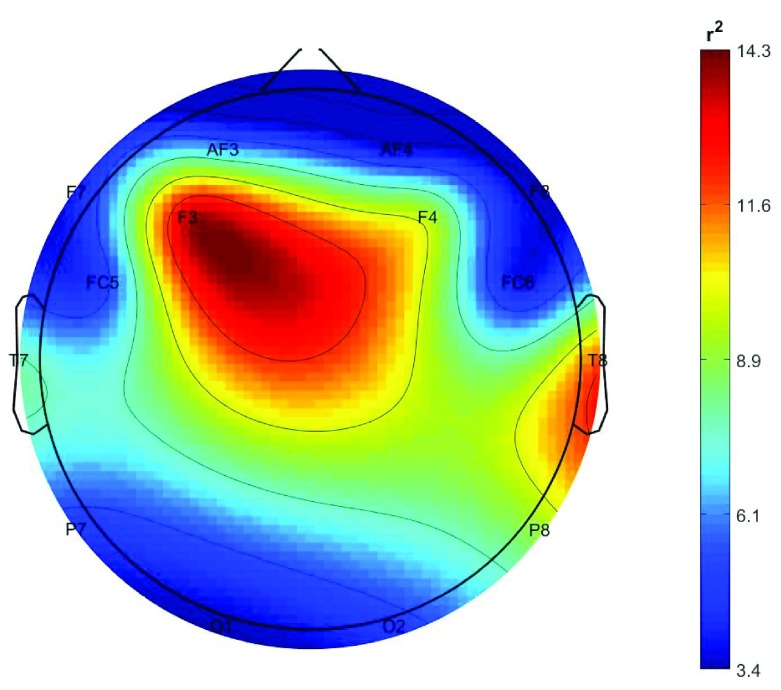
Topographic distribution of *r* ^2^ coefficients (stimulation vs non-stimulation discriminative power) in the stimulated participants.

**Figure 2.**
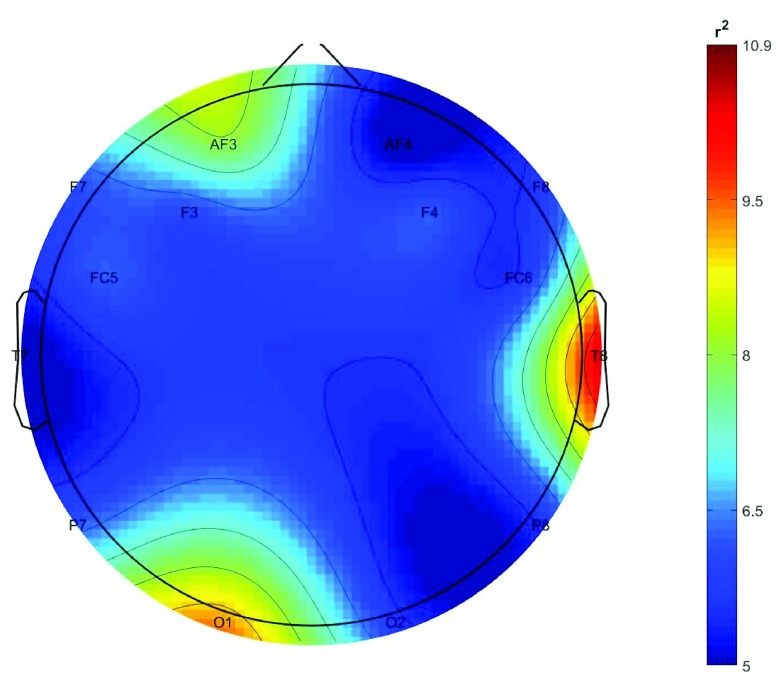
Topographic distribution of *r* ^2^ coefficients (stimulation vs non-stimulation discriminative power) in the non-stimulated participants.

Figure 1 and Figure 2 show the scalp distributions of *r ^2^* coefficients (expressed as percentage, normalized to the total score), representing the discriminative power between the periods of stimulation and no-stimulation (interstimulus intervals) of the features, for the stimulated and not-stimulated participants, respectively.

As it is shown in Figure 1, the class-discrimination (AVS vs nAVS) in the stimulated participants derives mostly from central and left frontal and right temporal electrodes, while in the non-stimulated participants their class is best discriminated by features from the right temporal and left occipital electrodes.

The percentages of the classification accuracy are reported in Table 1 and Table 2 for the stimulated and the non-stimulated participants, respectively.

**Table 1.** Percentages of the classifications for the stimulated participants. LD: linear discriminant classifier; Random: random classifier.

	LD	Random
**Iteration #1**	82.76	49.84
**Iteration #2**	82.95	50.58
**Iteration #3**	82.98	49.33
**Iteration #4**	82.78	49.64
**Iteration #5**	82.72	51.42
**Iteration #6**	82.58	49.91
**Iteration #7**	83.09	50.62
**Iteration #8**	82.86	51.10
**Iteration #9**	82.68	50.30
**Iteration #10**	83.00	50.34
**Mean**	82.84	50.31
**SD**	0.16	0.65

**Table 2.** Results of the classifications for the non-stimulated participants.

	LD	Random
**Iteration #1**	51.32	49.33
**Iteration #2**	51.09	49.81
**Iteration #3**	49.81	49.78
**Iteration #4**	51.06	48.96
**Iteration #5**	51.09	50.16
**Iteration #6**	50.33	50.95
**Iteration #7**	50.42	50.64
**Iteration #8**	50.95	49.72
**Iteration #9**	50.97	51.29
**Iteration #10**	50.34	50.30
**Mean**	50.74	50.09
**SD**	0.48	0.72

***Inferential statistics and parameters estimation.*** For the inferential statistics, we chose the comparison of percentage of correct discrimination between the stimulation and non-stimulation conditions, comparing each with the percentage obtained with the Random Classifier using a one-tailed paired t-test and a comparison using the Bayes Factor calculation using the default Cauchy prior value of .707. As a parameter estimation, we chose the measurement of Cohen’s effect size *d* and the relative confidence interval of 95%, all one-directional. All statistical analyses were conducted using Jasp software ( Jasp Team, 2018).

Results are presented in Table 3 below, while data from the Bayes Factor Robustness Check are shown in the Extended data.

**Table 3.** Inferential statistics and parameters estimation of the comparison of the percentage of correct classification of the periods of stimulation and non-stimulation between the linear discriminant and the random classifier.

	Stimulated participants	Non-stimulated participants
**t-test (** ***p*** **-value)**	154.84 (<.001)	2.06 (.035)
**Cohen’s** ***d*** **(lower 95% CI)**	48.9 (28.6)	.65 (.06)
**Bayes Factor _H1/H0_**	2.32 ^e13^	2.63

***Comment.*** While results for the stimulated partners are as expected, those observed from non-stimulated partners, from a statistical point of view indicate the presence of an EEG signal corresponding to the stimulation periods, even if the percentage of correct classification is not particularly high. Furthermore, the origins of these differences (see Figure 2) correspond to channels T8 and O1, which correspond to brain areas involved in processing auditory and visual information respectively.

### Second dataset

**Figure 3.**
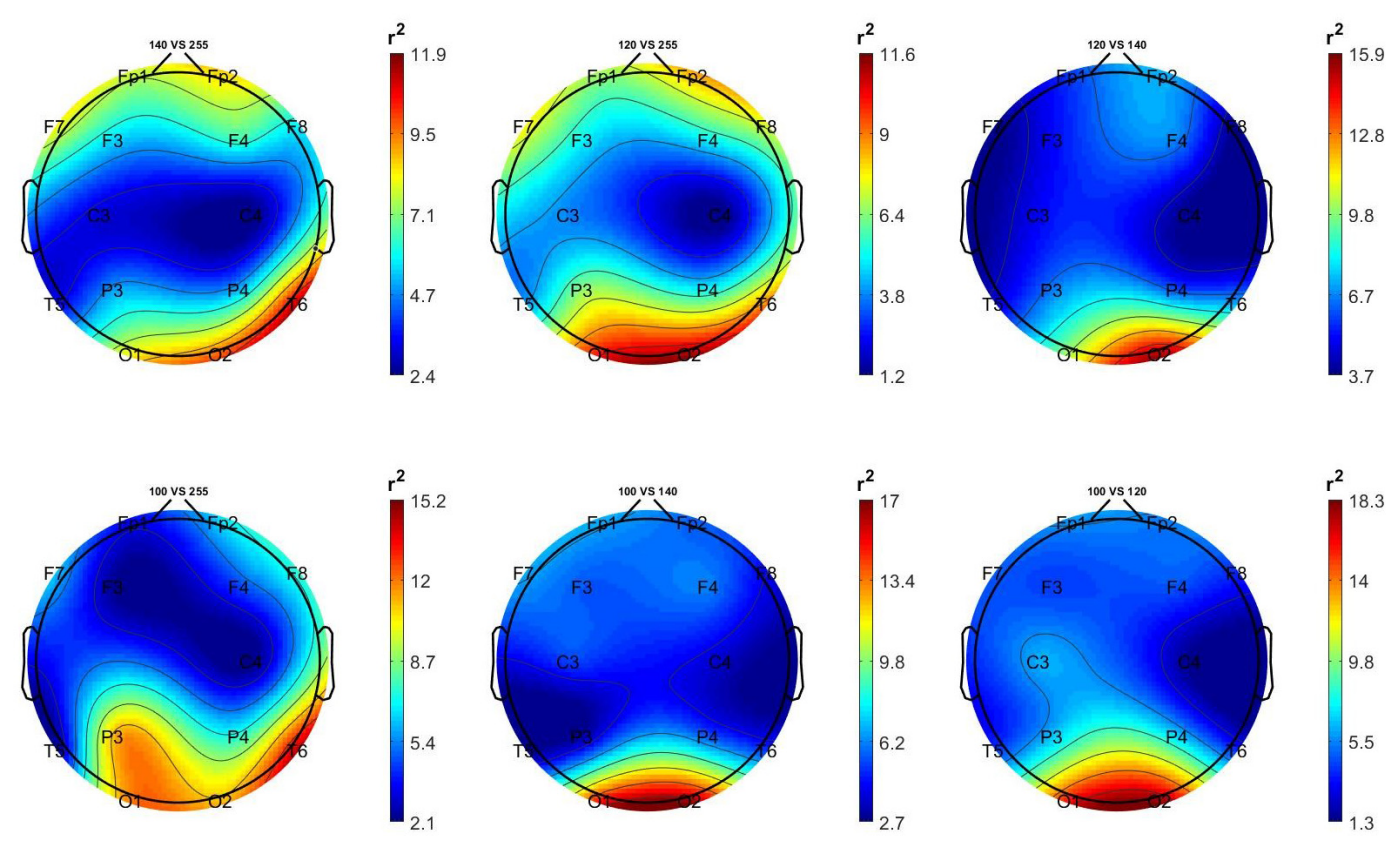
Topographic distribution of *r* ^2^ coefficients (stimulation vs non-stimulation discriminative power) of the stimulated participants. Top left: 14 Hz vs 0 Hz; top centre: 12 Hz vs 0 Hz; top right: 12 Hz vs 14 Hz; bottom left: 10 Hz vs 0 Hz; bottom centre: 10 Hz vs 14 Hz; bottom right; 10 Hz vs 12 Hz.

**Figure 4.**
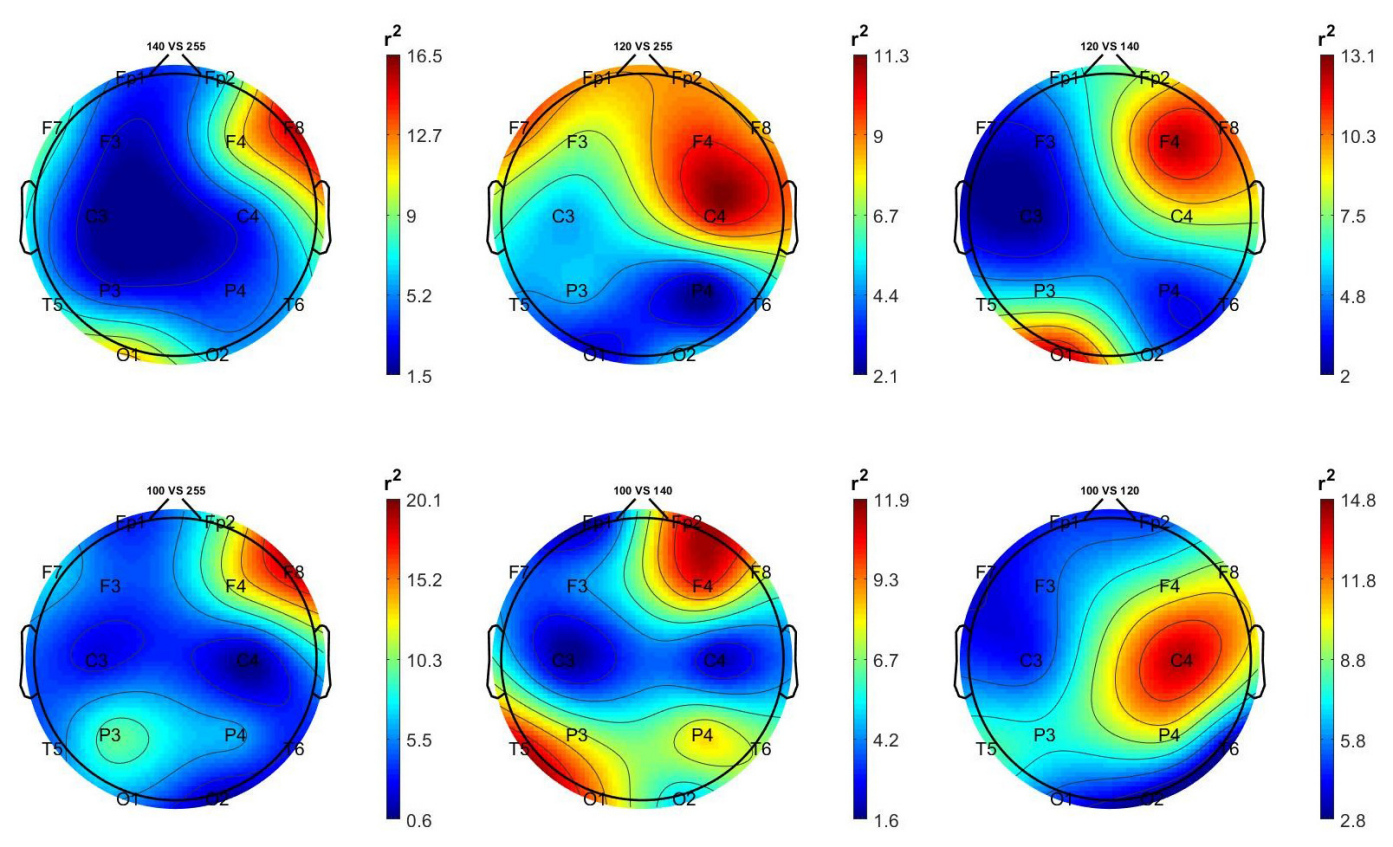
Topographic distribution of coefficients of *r* ^2^ (stimulation vs non-stimulation discriminative power) the non-stimulated participants. Top left: 14 Hz vs 0 Hz; top centre: 12 Hz vs 0 Hz; top right: 12 Hz vs 14 Hz; bottom left: 10 Hz vs 0 Hz; bottom centre: 10 Hz vs 14 Hz; bottom right; 10 Hz vs 12 Hz.

Figure 3 and Figure 4 show the scalp distributions of the mean *r* ^2^ coefficients (expressed as percentage, normalized to the total score), representing the discriminative power between the periods of stimulation and no-stimulation (interstimulus intervals) of the features, for each 2-class problem for each group (stimulated and non-stimulated pairs).

***Comment.*** As shown in Figure 3, in the stimulated participants the EEG activity generated by the three frequencies of stimulation is best distinguished from the 0 Hz class (non-stimulation) by the right occipital and temporal regions’ features.

In the non-stimulated participants ( Figure 4), the 10 Hz and 14 Hz are best distinguished from the 0 Hz class by frontal and frontocentral regions features.

***Percentages of classification accuracy.*** The percentages of correct classification for each comparison for each of the 10 cross-validation iterations and their descriptive statistics observed in the stimulated participants are presented in Table 4.

**Table 4.** Percentages of classification accuracies between the periods of stimulation and non-stimulation for the stimulated participants.

	10Hz vs 0Hz		12Hz vs 0Hz		14Hz vs 0Hz	
	LD	Random	LD	Random	LD	Random
**Iteration#1**	70.39	50.47	71.95	50.70	72.27	50.23
**Iteration#2**	71.25	49.30	72.50	47.66	72.42	49.38
**Iteration#3**	70.55	50.00	71.64	50.39	72.27	50.63
**Iteration#4**	69.84	49.45	73.05	51.56	71.72	50.31
**Iteration#5**	69.22	51.41	72.73	51.33	71.48	48.36
**Iteration#6**	69.84	50.31	73.05	51.88	71.72	50.94
**Iteration#7**	70.70	50.00	72.34	48.52	72.19	53.05
**Iteration#8**	70.08	48.91	72.03	48.83	72.11	50.63
**Iteration#9**	69.61	49.69	72.34	50.55	72.58	50.23
**Iteration#10**	69.61	50.16	72.19	51.41	72.11	52.42
**Mean**	**70.11**	49.97	**72.38**	50.28	**72.09**	50.62
**SD**	0.61	0.69	0.46	1.38	0.34	1.34

***Inferential statistics and parameters estimation.*** Similarly to the first dataset, for the inferential statistics we chose the comparison with the percentage of LD classifier discrimination between the three stimulation conditions at 10 Hz, 12 Hz, and 14 Hz, and the non-stimulation (0 Hz), comparing each with the percentages obtained from the Random Classifier using a paired one-tailed t-test and a comparison by way of the Bayes Factor calculation using the default Cauchy prior value of .707. As a parameter estimate, we chose Cohen’s *d* and the relative confidence interval of 95%, all one-directional. These results are presented in Table 5, while the Bayes Factor Robustness Check data are shown in the Extended data.

**Table 5.** Inferential statistics and parameters estimation of the comparison of the percentage of correct classification of the periods of stimulation and non-stimulation between the linear discriminant and the random classifier in the stimulated participants.

LD	Random	t-test	*p*	*d*	95%CI-Inf	BF _10_
10 Hz vs 0	10 Hz vs 0 r	56.95	< .001	18.01	10.92	7.45e ^9^
12 Hz vs 0	12 Hz vs 0 r	51.32	< .001	16.23	9.84	3.26e ^9^
14 Hz vs 0	14 Hz vs 0 r	51.91	< .001	16.42	9.95	3.57e ^9^

***Comment.*** As seen from the means, the percentage of correct classification of EEG activity between the non-stimulation and stimulation periods with the three frequency values is always greater than 70%, as confirmed by the inferential statistics results.

The percentages of correct classification of each comparison for each of the 10 cross-validation interactions and their descriptive statistics observed in the non-stimulated participants are presented in Table 6.

**Table 6.** Percentages of classification accuracies between the periods of stimulation and non-stimulation for the non-stimulated participants.

	10Hz vs 0Hz		12Hz vs 0Hz		14Hz vs 0Hz	
	LD	Random	LD	Random	LD	Random
**Iteration#1**	51.33	49.45	52.19	47.66	52.11	50.94
**Iteration#2**	50.78	50.47	50.16	48.83	51.64	50.31
**Iteration#3**	51.41	49.53	50.16	51.09	52.73	48.91
**Iteration#4**	52.27	48.67	51.56	49.84	53.44	48.75
**Iteration#5**	52.97	49.45	50.39	52.19	50.78	46.80
**Iteration#6**	50.47	50.39	49.69	47.58	50.16	51.09
**Iteration#7**	50.39	49.77	50.55	49.30	51.56	47.97
**Iteration#8**	52.42	52.50	48.52	50.08	52.42	49.14
**Iteration#9**	50.63	49.84	50.08	51.33	53.52	50.47
**Iteration#10**	49.06	50.47	51.25	51.09	50.78	48.28
**Mean**	**51.17**	50.05	**50.45**	49.90	**51.91**	49.27
**SD**	1.15	1.02	1.02	1.56	1.14	1.4

***Inferential statistics and parameters estimation.*** Results of inferential statistics and parameter estimates are shown in Table 7.

**Table 7.** Inferential statistics and parameters estimation of the comparison of the percentage of correct classification of the periods of stimulation and non-stimulation between the linear discriminant and the random classifier in the non-stimulated participants.

LD	Random	t-test	*p*	*d*	95%CI-Inf	BF _H1/H0_
**10 Hz vs 0**	10 Hz vs 0r	2.21	0.027	0.70	0.09	3.19
12 Hz vs 0	12 Hz vs 0r	0.87	0.202	0.27	-0.26	0.66
**14 Hz vs 0**	14 Hz vs 0r	4.97	< .001	1.57	0.75	101.98

***Comment.*** The most interesting result is that the activity related to two of the three stimulation frequencies, 10 Hz and 14 Hz, is classified above 50% with respect to the non-stimulation periods, even if in a more evident way for 14Hz. The fact that there are no differences between the 12Hz and the non-stimulation condition suggests that the base rhythm in non-stimulated participants is predominantly at 12Hz. Moreover, since the direct comparison between the activity corresponding to 10Hz and 14Hz, does not show differences with respect to the random condition (Mean = 50.2; SD = .75; Mean = 50.3; SD = 1.79), it suggests that in the non-stimulated participants, the EEG activity corresponding to these two frequencies either above or below 12Hz.

## Discussion

The machine-learning re-analyses of data from two studies relative to the distant correlation between EEG activity of participant pairs who were sensorially stimulated and participants who were connected to them only mentally, not only confirmed the existence of a correlation, as shown in the two studies from which they were taken, but also pointed out the degree of classification between stimulated and non-stimulated periods.

In the first dataset, the stimulation of EEG activity with luminous and auditory stimuli at 500Hz produced a slight perturbation also in that of the non-stimulated partners which allowed to distinguish it from the non-stimulation periods with a modest 50.74%. Even if the statistical comparison with the random classifier, slightly support a difference between the periods of the distant stimulation with respect to the period of distant non-stimulation, a difference of only 0.74%, cannot be considered very reliable.

In the second dataset, however, stimulation with frequencies of 10Hz and 14Hz, allowed to better distinguish this type of related perturbation, with percentages of 51.17% and 51.91% respectively, while for 12Hz stimulation, the discrimination was similar to the random classifier one.

Could these last results depend on a generic increase of alpha activity due to an increase relaxation or tiredness of non-stimulated participants during the task? We rule out this explanation because we observed differences with respect to the non-stimulation periods only for the 10 and 14 Hz, more pronounced for the last one (see percentage of classification, effect size and Bayes Factor values).

Even though, with hindsight, the choice to use frequencies that are within or close to the Alpha activity was not optimal, given that this was the dominant EEG activity in non-stimulated participants, the evidence that these are distinguishable even in the EEG activity of non-stimulated participants opens interesting possibilities, should this research path be continued in terms of practical applications.

Information regarding the potential neural sources of these signals obtained from this study must also be validated with confirmatory studies and also with more sophisticated equipment than those used by us.

From a theoretical point of view, while we can safely reject the theory that the observed activity in non-stimulated participants is due to a traditional sensorial transmission (albeit weak) from the stimulated participants’ EEG activity, we as yet do not have enough information to determine if this correlation – which does not necessarily involve transmission – has quantum-like properties like the physical particles studied in quantum mechanics. Indeed, to determine this it would, first of all, entail closing a series of loopholes, as was done in quantum mechanics to reject the theory that distant connection used the laws of classical physics ( Giustina *et al*., 2015; Shalm *et al*., 2015). For example, the loophole showing the impossibility of communication at the speed of light between participant pairs would need closing.

Pending a better understanding of the laws regulating the correlation between the EEG activity of two mentally-connected participants, we believe that data obtained so far in this field, including that of other researchers, clearly show that if classification algorithms of signals down to the level of single participants are perfected, along with an online instead of the offline classification, the road towards practical use of mental telecommunication can be opened, even if for now it is based on binary symbolic codes.

Mental telecommunication, not just those based on electromagnetic waves but also those of the future based on quantum technologies, have the advantage of not requiring repeaters or security measures and would certainly be beneficial from an economical point of view.

## Data availability

### Underlying data

*First dataset:* Figshare: EEG correlates of social interaction at distance, http://dx.doi.org/10.6084/m9.figshare.1466876 ( Tressoldi, 2016).

*Second dataset:* Figshare: Brain-to-Brain interaction at a distance: a global or differential relationship? https://doi.org/10.6084/m9.figshare.5132647.v7 ( Tressoldi *et al*., 2018)

Data are available under the terms of the Creative Commons Attribution 4.0 International license (CC-BY 4.0).

### Extended data

Dataset 1: Stimulated participants. Bayes Factor Robustness Check; Non-stimulated participants: Bayes Factor Robustness Check

Dataset 2: Non-stimulated participants. Bayes Factor Robustness Check: 10 Hz vs 0 Hz; Bayes Factor Robustness Check: 14 Hz vs 0 Hz

Both datasets are available on Figshare: Brain-to-Brain interaction at a distance: a global or differential relationship? https://doi.org/10.6084/m9.figshare.5132647.v7 ( Tressoldi *et al*., 2018)

Data are available under the terms of the Creative Commons Attribution 4.0 International license (CC-BY 4.0).
