# Supplementary material for: EEG correlation at a distance: A re-analysis of two studies using a machine learning approach
Source: F1000Res. 2019 Mar 29;8:43. Originally published 2019 Jan 10. [Version 2] doi: 10.12688/f1000research.17613.2 (PMC6713066; doi:10.12688/f1000research.17613.2)
Supplement: Peer review history

# Peer review: Reviewer response for version 2

**DOI:** 10.5256/f1000research.20515.r46491

**Published:** 2019-08-27

**Authors:** Schubert R. Carvalho

**Affiliations:** 1 Vale Institute of Technology (ITV), Belém, Brazil

The paper is scientifically sound, and the authors made improvements from the first submission and adequated handle my raised concerns of the first submission.

I confirm that I have read this submission and believe that I have an appropriate level of expertise to confirm that it is of an acceptable scientific standard.

# Peer review: Reviewer response for version 2

**DOI:** 10.5256/f1000research.20515.r46492

**Published:** 2019-04-30

**Authors:** Arnaud Delorme

**Affiliations:** 1 Brain and Cognition Research Centre, CNRS, Paul Sabatier University, Toulouse, France

In experiment 2, the experimenters didn't mix randomly the three frequencies (they were presented in blocks). However, the order of the blocks was different in the different subjects, so this mitigates my original comment of the absence of randomization.

I confirm that I have read this submission and believe that I have an appropriate level of expertise to confirm that it is of an acceptable scientific standard.

# Peer review: Reviewer response for version 1

**DOI:** 10.5256/f1000research.19259.r42948

**Published:** 2019-03-21

**Authors:** Schubert R. Carvalho

**Affiliations:** 1 Vale Institute of Technology (ITV), Belém, Brazil

The study presented in this paper is challenging and can be fascinating; it aims to verify whether or not two participants are only connected mentally without using sensory organs. To check this connection, the authors decided to use a machine learning approach. They used a linear classifier and framed the problem as a classification task. The methodology was validated on two public datasets that both used EEG signals as a measure for brain activity. 

 The methodology is simple to grasp and understand. The main steps of the methodology can be reproduced. However, it seems the authors get lost in the results section.  

 The study design can be improved to verify mental telecommunication (whether or not two brains are connected). Figures 1 and 2 show the stimulated and non-stimulated brains of the participants. One good comparison, was showing the differences between the brains at rest. If the goal is to verify if two brains are connected, one observation that is missing is the brain at rest vs. brain at stimulus for both groups. For example, if the two brains (two groups) are connected, then the classification between both should be random.

 The results shown in table 1 and 2 sound obvious. Table 1 shows the accuracy of the stimulated and non-stimulated brain (stimulated participants group), the classes show different patterns of activity, as measured by the high classification accuracy compared to the data of table 2. However, the comparison that is missing is between the data of the stimulated and non-stimulated subjects during the stimulation period. So, if the classification accuracy is at random, it suggests a brain connection. Mental telecommunication is the primary goal of this paper after all.

 The same principle of analysis was done for dataset 2 (Results section: Second dataset). It is not possible to conclude mental telecommunication from this analysis, as shown in table 4. So, one interesting experiment that could be done to verify mental telecommunication would be to analyze the non-stimulated group data before and during the stimulation, to see if the mental states are separable as in the stimulated group. If this analysis is in the paper, it is not clear. 

 In the second paragraph of the discussion section, the authors argue that a modest classification accuracy of 50.74% allowed to distinguish it from the non-stimulation condition. From a machine learning point of view, this is considered a random classification. Moreover, this study (as it is in the moment) did not show evidence (machine learning results) for mental telecommunication — the same for the third paragraph (second dataset).

I confirm that I have read this submission and believe that I have an appropriate level of expertise to confirm that it is of an acceptable scientific standard, however I have significant reservations, as outlined above.

# Response

**Published:** 2019-03-25

**Authors:** Patrizio Tressoldi

**Affiliations:** Dipartimento di Psicologia Generale, Università di Padova, Italy

"The study design can be improved to verify mental telecommunication (whether or not two brains are connected). Figures 1 and 2 show the stimulated and non-stimulated brains of the participants. One good comparison, was showing the differences between the brains at rest. If the goal is to verify if two brains are connected, one observation that is missing is the brain at rest vs. brain at stimulus for both groups. For example, if the two brains (two groups) are connected, then the classification between both should be random."

**Reply: what Figures 1 and 2 show, is precisely the spatial distribution of the features (extracted with the method described in the “Features selection” section) ranked by their discriminative power (r2 coefficients) for the stimulation versus the non-stimulation periods, in the stimulated (Figure 1) and the non-stimulated participants (Figure 2).**

**The same information is presented in the Figures 3 and 4 with respect to the second dataset.**

**We now have clarified this in the Figures’ captions.**

"The results shown in table 1 and 2 sound obvious. Table 1 shows the accuracy of the stimulated and non-stimulated brain (stimulated participants group), the classes show different patterns of activity, as measured by the high classification accuracy compared to the data of table 2. However, the comparison that is missing is between the data of the stimulated and non-stimulated subjects during the stimulation period. So, if the classification accuracy is at random, it suggests a brain connection. Mental telecommunication is the primary goal of this paper after all."

**Reply:** **your suggestion that a random classification accuracy comparing the data of the stimulated and non-stimulated subjects during the stimulation period, should be the critical proof of a “brain-to-brain” connection at distance should be valid postulating a similar EEG activity in the stimulated and non-stimulated subjects.**

**In the new paragraph “Experimental hypotheses” on page 5, we now have clarified that this effect is not possible because there cannot be a transmission or connection of the “physical” characteristics of the EEG activity trigger by the visual-auditory stimulations, between the stimulated and non-stimulated subjects, but only a still undefined type of information which weakly affect the EEG activity of the non-stimulated subjects.**

"The same principle of analysis was done for dataset 2 (Results section: Second dataset). It is not possible to conclude mental telecommunication from this analysis, as shown in table 4. So, one interesting experiment that could be done to verify mental telecommunication would be to analyze the non-stimulated group data before and during the stimulation, to see if the mental states are separable as in the stimulated group. If this analysis is in the paper, it is not clear."

**Reply: we now have clarified that the data and the statistics reported in Tables are precisely those related to the comparison between the periods of stimulation and the interstimulus (no-stimulation) periods.**

"In the second paragraph of the discussion section, the authors argue that a modest classification accuracy of 50.74% allowed to distinguish it from the non-stimulation condition. From a machine learning point of view, this is considered a random classification. Moreover, this study (as it is in the moment) did not show evidence (machine learning results) for mental telecommunication — the same for the third paragraph (second dataset)."

**Reply: we agree that the marginal 0.74% difference, even if statistically different from the random classification, cannot be considered reliable (now added in the discussion).**

**However, the differences observed in the second dataset, even if very modest according to the standards used in the machine learning community, it is sufficient to support the hypothesis that the EEG activity of the non-stimulated participants is in some way related to those of the stimulated one. As commented in the “Discussion” section, we think that this hypothesis can be further supported improving our machine learning algorithms. This is our optimistic opinion of course.**

# Peer review: Reviewer response for version 1

**DOI:** 10.5256/f1000research.19259.r42945

**Published:** 2019-01-30

**Authors:** Arnaud Delorme

**Affiliations:** 1 Brain and Cognition Research Centre, CNRS, Paul Sabatier University, Toulouse, France

This article intends to show that machine learning methods might be able to pick on subtle EEG activity and perform classification above chance level and show true EEG correlation at a distance. The article is well written and clear.

 I was able to download the data and reproduce the results of experiment 2. I am not sure about the first experiment as the processing script for that experiment seems to be missing. The data processing, machine learning and cross-validation scheme seemed straightforward and appropriate. It is still possible that by trying many machine learning approaches, one could obtain positive results just by chance. This should be mentioned in the discussion as a potential limitation. 

 The most interesting result is that participants who are not exposed to stimuli nevertheless have a response to them that the classifier can pick up above chance expectations. One important caveat I have seen in the experimental design of experiment 2 is that the stimuli are not randomly intermixed. Instead there is a sequence of one stimulus type and then another one, etc. As subjects perform the experiment, their EEG changes (for example they might get tired as the experiment progresses which could affect their alpha brain waves). So one might have more alpha for one stimulus type simply because a given stimulus type is presented first. The classifier can then pick up on that. The sequence of stimuli should have been randomized for that reason. In other words, since stimuli of a given type are grouped, the classifier might simply pick up on the fact that some epochs belong to the beginning of the experiments, and some others belong to the end.

 If my assessment is correct, I would remove the second experiment from the manuscript. Once I get the processing script for the first experiment, I would be able to assess potential pitfalls.

 Abstract: the abstract should mention statistical results.

I confirm that I have read this submission and believe that I have an appropriate level of expertise to state that I do not consider it to be of an acceptable scientific standard, for reasons outlined above.

# Response

**Published:** 2019-01-30

**Authors:** Patrizio Tressoldi

**Affiliations:** Dipartimento di Psicologia Generale, Università di Padova, Italy

Sorry for missing the syntax for reproducing our machine learning analyses on the first dataset. We have just uploaded it "MachineLearningSyntaxExperiment1" on https://doi.org/10.6084/m9.figshare.5132647.v8

We are also pleased you were able to reproduce our analyses on the dataset 2 and appreciated their appropriateness.

As to your main concerns that the results observed in the participants who were not exposed to stimuli, were due to "alpha activity of one stimulus type simply because a given stimulus type is presented first and they might get tired as the experiment progresses which could affect their alpha brain waves", we consider this interpretation disputable given the following reasons:

1. The stimulus types (10, 12, 14 Hz) were presented randomly. How is it possible to obtain a fair good level of correct classification of all three stimulus types with respect to the non-stimulation periods in the stimulated participants and in two out of three stimulus types in the non-stimulated participants? Let us suppose an almost equal distribution of the initial presentation of the three stimulus types among the 20 pairs of participants, how is it possible to discriminate their corresponding EEG activity with respect to the non-stimulation one if based on just the type of the first stimulus?
2. In the non-stimulated participants we observed differences with respect to the non-stimulation periods only for the 10 and 14 Hz, more pronounced for the last one (see percentage of classification, effect size and Bayes Factor values). How is it possible to justify these differences postulating a common tiring effect?
